# Beyond Pancreatic Ductal Adenocarcinoma: A Narrative Review of the Radiological and Endoscopic Features of Solid Pancreatic Neoplasms

**DOI:** 10.7759/cureus.119145

**Published:** 2026-11-05

**Authors:** Chorouq Mehdaoui, Claire Billioud

**Affiliations:** 1 Hepatogastroenterology, Centre Hospitalier Universitaire Mohammed VI, Oujda, MAR; 2 Gastroenterology, Hôpital de la Croix-Rousse, Lyon, FRA

**Keywords:** differential diagnosis, endoscopic ultrasound, immunohistochemical findings, pancreatic acinar cell carcinoma, pancreatic ductal adenocarcinoma, pancreatic neuroendocrine neoplasms, radiological findings, solid pancreatic neoplasms

## Abstract

Solid pancreatic neoplasms comprise a heterogeneous group of tumors with distinct histopathological characteristics, biological behaviors, and therapeutic implications. Although pancreatic ductal adenocarcinoma (PDAC) is the predominant malignancy, non-ductal neoplasms, including pancreatic acinar cell carcinoma (PACC), pancreatic neuroendocrine tumors (PanNETs), solid pseudopapillary neoplasms (SPNs), and pancreatoblastoma, represent important diagnostic alternatives. This narrative review aims to summarize and compare the radiological and endoscopic features of solid pancreatic neoplasms, emphasizing their differential diagnosis and the contribution of endoscopic ultrasound (EUS)-guided tissue acquisition to diagnostic characterization. A narrative review of the literature was conducted, focusing on studies addressing CT, MRI, conventional and contrast-enhanced EUS, elastography, and EUS-guided tissue acquisition in the characterization and differential diagnosis of solid pancreatic tumors.

PDAC typically demonstrates infiltrative growth and reduced enhancement, whereas non-ductal neoplasms may exhibit well-defined margins and variable vascular patterns. PACC may present as a well-circumscribed mass with heterogeneous enhancement and areas of cystic degeneration, while PanNETs frequently demonstrate hypervascularity, and SPNs often exhibit mixed solid and cystic components. However, substantial imaging overlap limits reliable histological differentiation. Beyond the morphological and vascular characterization provided by EUS, EUS-guided fine-needle biopsy (EUS-FNB), combined with histological and immunohistochemical analysis, plays a crucial role in establishing a definitive diagnosis, particularly for uncommon neoplasms. Emerging technologies, including radiomics and artificial intelligence (AI), offer promising opportunities for improving diagnostic characterization. Accurate differentiation of solid pancreatic neoplasms requires an integrated approach combining cross-sectional imaging, EUS, and histopathological assessment. Recognizing characteristic imaging patterns and understanding their limitations may improve diagnostic accuracy and guide appropriate tissue acquisition and therapeutic decision-making.

## Introduction and background

Solid pancreatic neoplasms comprise a heterogeneous group of tumors with distinct histopathological characteristics, biological behaviors, and prognostic implications [1,2]. Although pancreatic ductal adenocarcinoma (PDAC) is the most common malignant pancreatic neoplasm, several other entities should also be considered in the differential diagnosis of solid pancreatic masses. These include pancreatic neuroendocrine neoplasms (PanNENs), pancreatic acinar cell carcinoma (PACC), solid pseudopapillary neoplasms (SPNs), pancreatoblastoma, and rare histological variants of pancreatic carcinoma [1,2].

Accurate preoperative differentiation remains challenging because different histopathological subtypes may share overlapping clinical and imaging features [2,3]. Even so, imaging features such as tumor margins, enhancement patterns, internal architecture, calcifications, and pancreatic duct abnormalities may provide valuable clues to the underlying histopathological subtype [2,3]. Recognizing these differences is clinically important because biological behaviors vary and call for distinct diagnostic and therapeutic approaches [1]. For instance, differentiating PDAC from well-differentiated PanNETs or SPNs may influence subsequent diagnostic investigations and clinical management, given their distinct histopathological characteristics and biological behaviors [1].

Multiphasic CT and MRI contribute to the detection, characterization, and assessment of local tumor extent and resectability, while endoscopic ultrasound (EUS) provides complementary information through high-resolution imaging and EUS-guided tissue acquisition [3,4]. Advanced EUS techniques, including contrast-enhanced EUS and elastography, may further improve lesion characterization by assessing tumor vascularity and tissue stiffness [2]. EUS-guided tissue acquisition, particularly fine-needle biopsy (EUS-FNB), enables histopathological evaluation and ancillary testing, including immunohistochemical analysis, when tissue confirmation is indicated, which contributes to accurate tumor classification [4,5].

This narrative review aims to critically summarize and compare the radiological and endoscopic features of the major histopathological types of solid pancreatic neoplasms, while highlighting their distinguishing characteristics, diagnostic overlap, and the role of EUS-guided tissue acquisition and immunohistochemical analysis in establishing an accurate histopathological diagnosis.

## Review

Methods

Literature Search Strategy

This narrative review focuses on the endoscopic and radiological characteristics of solid pancreatic neoplasms, with particular emphasis on their differential diagnosis by histopathological subtype. The literature search was performed from December 2025 to March 2026, with a subsequent update in October 2026, using PubMed/MEDLINE. Individual search phrases included “pancreatic acinar cell carcinoma”, “pancreatic ductal adenocarcinoma”, “ultrasonography of pancreatic neoplasms”, “imaging of pancreatic neoplasms”, “diagnosis of pancreatic neuroendocrine tumors”, “pancreatoblastoma”, “endoscopic ultrasound of pancreatic neoplasms”, “magnetic resonance imaging of pancreatic neoplasms”, and “histopathological features of pancreatic neoplasms”. Full-text articles were accessed through publisher platforms, including ScienceDirect, when available.

Publications from 2023 to 2026 were prioritized to incorporate recent advances in diagnostic imaging and endoscopic techniques, while earlier landmark studies were included when relevant. Priority was given to clinical practice guidelines, systematic reviews, meta-analyses, and original studies addressing CT, MRI, conventional and contrast-enhanced EUS, elastography, and EUS-guided tissue acquisition. Articles were reviewed individually and selected based on their recency, clinical relevance, and contribution to the differential diagnosis of solid pancreatic neoplasms. Selection was based on clinical applicability rather than predefined systematic inclusion and exclusion criteria. The findings were synthesized narratively, with emphasis on the overlapping and distinguishing radiological, endosonographic, and histopathological characteristics of different pancreatic tumor subtypes. No formal risk-of-bias assessment or quantitative meta-analysis was performed.

Histopathological classification of solid pancreatic neoplasms

Solid pancreatic neoplasms comprise a heterogeneous group of tumors characterized by distinct cellular differentiation, morphological patterns, and molecular profiles, which contribute to their variable biological behaviors and diagnostic presentations [6,7]. PDAC is the predominant malignant epithelial tumor of the pancreas, typically characterized by infiltrative, irregular glandular structures embedded in a dense desmoplastic stroma [8]. Beyond conventional PDAC, several histological variants are recognized, including adenosquamous carcinoma, colloid carcinoma, and undifferentiated carcinoma, which may exhibit distinct morphological and biological characteristics [8].

PanNENs are divided into well-differentiated pancreatic neuroendocrine tumors (PanNETs), graded G1, G2, or G3 according to mitotic activity and the Ki-67 proliferation index, and poorly differentiated pancreatic neuroendocrine carcinomas (PanNECs), which constitute a separate, high-grade malignant category [1]. Histologically, PanNETs typically consist of relatively uniform cells with round to oval nuclei, arranged in nests, trabeculae, or rosette-like structures [9]. 

PACC represents a distinct exocrine malignancy characterized by acinar differentiation, with tumor cells displaying granular eosinophilic cytoplasm and prominent nucleoli. Its diagnosis is supported by the expression of pancreatic exocrine enzymes, particularly trypsin and chymotrypsin [10,11]. Tumors exhibiting combined acinar and neuroendocrine differentiation have also been described, highlighting the potential histological overlap between these entities [12].

SPNs are uncommon pancreatic tumors characterized by a combination of solid and pseudopapillary structures, often associated with hemorrhagic and cystic degeneration [13]. Histologically, pseudopapillary formations, hyaline globules, and cholesterol clefts may provide useful diagnostic clues [14]. These neoplasms are frequently associated with alterations in the Wnt/β-catenin signaling pathway, resulting in abnormal nuclear accumulation of β-catenin [15]. Although generally classified as low-grade malignant neoplasms, a minority may demonstrate aggressive behavior [15].

Pancreatoblastoma is another rare epithelial neoplasm, predominantly encountered in children, characterized by a hypercellular proliferation of primitive cells exhibiting acinar differentiation and distinctive squamoid nests, which represent a defining histopathological feature [6]. These histopathological differences provide the biological basis for the heterogeneous radiological and endoscopic appearances of solid pancreatic neoplasms. However, overlapping morphological characteristics, particularly between acinar cell carcinoma, neuroendocrine neoplasms, and pancreatoblastoma, may complicate definitive classification and require complementary immunohistochemical analysis [16,17].

Radiological evaluation of solid pancreatic neoplasms

Cross-sectional imaging plays a central role in the detection, characterization, and staging of solid pancreatic neoplasms, with multiphasic CT and MRI providing complementary information on tumor morphology, vascularity, ductal involvement, and relationships with adjacent structures [16,18]. Although PDAC represents the principal diagnostic consideration when a solid pancreatic mass is identified, several less common neoplasms may demonstrate overlapping imaging features, making accurate histological differentiation challenging [1,2].

Multiphasic CT

Pancreatic-protocol contrast-enhanced CT is a cornerstone of the initial evaluation of solid pancreatic tumors, allowing assessment of lesion size, enhancement characteristics, pancreatic and biliary ductal changes, vascular involvement, and metastatic disease [16,18]. Conventional PDAC typically appears as a poorly defined, hypoenhancing mass with an infiltrative growth pattern, frequently associated with upstream pancreatic duct dilation, parenchymal atrophy, and, when located in the pancreatic head, biliary obstruction [1,16]. In contrast, PACC often presents as a relatively well-circumscribed, expansible or exophytic mass, usually hypoenhancing relative to the surrounding pancreatic parenchyma with internal heterogeneity related to cystic degeneration or necrosis, particularly in larger tumors. Internal calcifications may occasionally be observed [17,19].

In their radiological description of PACC, Tatli et al. (2005) reported well-defined margins in 10 of 11 patients (90.9%) and exophytic growth in nine of 11 patients (81.8%), with cystic areas predominantly observed in larger tumors [19]. These features may help distinguish PACC from the more infiltrative appearance of conventional PDAC. Nevertheless, these characteristics are not pathognomonic, and PACC may occasionally exhibit invasive features or associated ductal abnormalities, limiting the reliability of morphology alone [18].

PanNENs, particularly well-differentiated tumors, frequently demonstrate well-defined margins and pronounced arterial-phase enhancement reflecting their rich vascular supply [16]. However, larger lesions and higher-grade neoplasms may show heterogeneous or reduced enhancement, necrosis, and invasive features, creating substantial overlap with PACC and PDAC [1,3].

SPNs are typically well-demarcated, expansile tumors with variable proportions of solid and cystic components, often associated with a peripheral capsule, hemorrhagic degeneration, and occasionally calcifications [3,16]. Their enhancement is generally less pronounced during the arterial phase than that of well-differentiated PanNENs, although predominantly solid SPNs may remain difficult to differentiate from other pancreatic neoplasms [3].

MRI

MRI provides additional tissue characterization through T1- and T2-weighted sequences, diffusion-weighted imaging, dynamic contrast-enhanced acquisitions, and magnetic resonance cholangiopancreatography (MRCP), particularly when CT findings are equivocal or ductal anatomy requires further assessment [18,20]. PACC generally demonstrates low signal intensity on T1-weighted images and variable, often heterogeneous signal intensity on T2-weighted images, ranging from isointensity to hyperintensity relative to the surrounding pancreatic parenchyma [20]. Large PACC lesions may contain areas of necrosis or hemorrhage, and their relatively circumscribed appearance can resemble that of PanNENs or SPNs [19,20].

MRI is particularly valuable in the evaluation of SPNs because it can demonstrate intratumoral hemorrhage, cystic degeneration, and a peripheral capsule more clearly than CT in selected cases [3]. In a retrospective study involving 39 patients with SPNs and 127 patients with PanNENs, Khristenko et al. (2024) identified the presence of a capsule, absence of arterial hyperenhancement, and younger patient age as independent predictors favoring SPN over PanNEN [3].

PanNENs often exhibit T1 hypointensity, variable T2 hyperintensity, and avid enhancement following contrast administration, although these findings are influenced by tumor differentiation, size, and biological aggressiveness [3,16]. Diffusion-weighted imaging may provide complementary information about lesion conspicuity and tissue characteristics, but substantial overlap between tumor subtypes limits its ability to establish a definitive histological diagnosis in isolation [18].

Morphological and Ductal Features in Differential Diagnosis

Beyond enhancement characteristics, tumor margins, growth pattern, size, and associated ductal changes are important elements in the radiological differential diagnosis of solid pancreatic neoplasms [16,17]. PDAC commonly demonstrates an infiltrative appearance with pancreatic duct obstruction and upstream parenchymal atrophy, whereas PACC more frequently exhibits expansive growth and may cause relatively limited ductal dilatation despite considerable tumor size [18,19]. Nevertheless, ductal dilatation does not exclude PACC, and the absence of ductal obstruction does not reliably rule out PDAC, particularly in small or atypically located lesions [18].

The distinction between PACC and PanNENs can be especially difficult when both present as well-circumscribed solid masses, although marked arterial hyperenhancement generally favors a well-differentiated PanNEN, while larger size, heterogeneous enhancement, and extensive necrotic components may raise suspicion for PACC [17,18]. Similarly, SPNs may mimic PACC or PanNENs when their cystic or hemorrhagic components are limited, but the combination of a capsule, relatively weak arterial enhancement, and characteristic clinical demographics can improve diagnostic orientation [3].

Other uncommon solid pancreatic lesions, including pancreatoblastoma, pancreatic lymphoma, metastases, and rare epithelial or mesenchymal neoplasms, may also present with overlapping morphological characteristics and should be considered when the imaging appearance is atypical for conventional PDAC [1,2].

Limitations of Cross-Sectional Imaging

Despite the diagnostic value of CT and MRI, considerable overlap persists among solid pancreatic neoplasms, particularly between PACC, atypical PanNENs, predominantly solid SPNs, and uncommon PDAC variants [2,18]. Furthermore, tumor size, necrosis, fibrosis, and histological heterogeneity may modify otherwise characteristic imaging appearances, reducing the specificity of individual radiological signs [1,17]. Consequently, cross-sectional imaging should be interpreted in conjunction with clinical findings and, when indicated, endoscopic ultrasound-guided tissue acquisition to achieve accurate tumor classification and guide management [1,4].

Endoscopic ultrasound evaluation and EUS-guided tissue acquisition

EUS plays a central role in the characterization of solid pancreatic neoplasms by providing high-resolution assessment of lesion morphology, internal architecture, ductal relationships, and vascular involvement [21]. Advanced techniques, including contrast-enhanced harmonic EUS (CH-EUS) and EUS elastography, provide complementary information on tumor vascularity and tissue stiffness, while EUS-guided tissue acquisition enables histopathological confirmation and accurate tumor classification when indicated [22,23].

Conventional EUS Features

PDAC typically appears as an irregular, hypoechoic mass with poorly defined margins, frequently associated with upstream pancreatic duct dilatation or vascular involvement [21]. In a multicenter study involving 761 patients, irregular margins and ductal dilatation were significant predictors of PDAC, whereas PanNENs more frequently demonstrated regular margins and homogeneous echotexture [21].

PACC may appear as a well-demarcated, hypoechoic or occasionally isoechoic or slightly hyperechoic mass, with lobulated contours and variable internal heterogeneity and occasional internal calcifications [24]. Larger lesions may contain necrotic or degenerative areas, resulting in morphological overlap with PanNENs and other non-ductal tumors [11]. However, conventional EUS findings of PACC remain poorly documented, and no specific sonographic features have been established to reliably distinguish this tumor from other solid pancreatic neoplasms [24].

SPNs exhibit variable EUS appearances, ranging from predominantly solid lesions to heterogeneous masses with cystic and hemorrhagic components [25]. In an international multicenter study involving 106 patients, 55.7% of SPNs were predominantly solid, illustrating the limitations of relying on cystic degeneration as a distinguishing feature [25].

Contrast-Enhanced Harmonic EUS

CH-EUS provides real-time visualization of tumor microvascularization and may improve the characterization of solid pancreatic lesions beyond conventional grayscale imaging [22]. PDAC typically exhibits hypoenhancement relative to the surrounding pancreatic parenchyma, whereas well-differentiated PanNENs more frequently demonstrate hyperenhancement, although atypical vascular patterns may occur [26].

Specific enhancement patterns have also been investigated for differentiating SPNs from PanNENs [27]. In a comparative study including 29 SPNs and 77 nonfunctional PanNENs, Kataoka et al. (2022) described the alveolus nest sign, characterized by small nonenhancing spaces within enhancing tumor tissue, as a feature favoring SPNs. This sign was observed in 18 of 25 SPNs (72%) and four of 60 PanNENs (6.7%) among patients who underwent CH-EUS, suggesting that internal vascular architecture may contribute to the differential diagnosis beyond overall enhancement intensity [27].

In contrast, CH-EUS findings in PACC remain insufficiently characterized, with available evidence largely derived from small series and individual reports [24]. The enhancement pattern may vary according to the microscopic structure of the tumors, particularly their vascularity, cellularity, and degenerative changes [24]. Heterogeneous or variable enhancement has been described, but no sufficiently validated vascular pattern reliably distinguishes PACC from other non-ductal pancreatic neoplasms [2].

EUS Elastography

EUS elastography provides complementary information on the mechanical properties of pancreatic lesions by assessing tissue stiffness, which may contribute to the differentiation of malignant and benign pancreatic masses [26]. In a recent retrospective study involving 102 patients, including 74 patients with PDAC, 15 with benign pancreatic lesions, and 13 with normal pancreatic tissue, Wang et al. (2025) evaluated several quantitative elastographic parameters, including the strain ratio, fat-lesion strain ratio, and mean strain histogram, for the identification of PDAC. Among patients with pancreatic lesions, the strain ratio demonstrated the highest individual diagnostic performance (AUC = 0.800), while the combination of all three parameters slightly improved diagnostic accuracy (AUC = 0.826). These findings suggest that quantitative elastography may provide complementary diagnostic information, although the study primarily assessed PDAC rather than the differentiation of individual histological subtypes of solid pancreatic neoplasms [28].

Beyond lesion characterization, EUS elastography has also been investigated as a complementary technique for guiding tissue acquisition toward suspicious areas. However, the available evidence does not consistently demonstrate an improvement in diagnostic performance compared with conventional EUS-guided fine-needle aspiration [26]. However, elastographic findings may overlap among different pancreatic neoplasms, limiting their ability to establish a specific histological diagnosis. In PACC, available evidence remains limited, and elastographic appearances may resemble those observed in PDAC [24]. Therefore, EUS elastography should be considered a complementary technique rather than a substitute for histopathological confirmation [26].

EUS-Guided Tissue Acquisition

EUS-guided tissue acquisition is particularly important when imaging cannot distinguish pancreatic neoplasms with different prognostic and therapeutic implications [1]. Although EUS-guided fine-needle aspiration (EUS-FNA) remains an established diagnostic technique, EUS-FNB provides tissue cores that facilitate histological evaluation, immunohistochemistry, and selected molecular analyses [5]. In a meta-analysis of 12 prospective studies, including seven randomized controlled trials and five cohort studies, Yao et al. (2024) reported higher diagnostic accuracy (risk difference (RD) for EUS-FNB: -0.08; 95% CI: -0.15 to -0.01) and specimen adequacy (RD: -0.08; 95% CI: -0.15 to -0.02) with EUS-FNB than with EUS-FNA, together with fewer required needle passes (mean difference: 0.42; 95% CI: 0.12-0.73, favoring EUS-FNB) and comparable complication rates [23]. These findings support the increasing preference for FNB in the evaluation of solid pancreatic masses [23].

Recent research has also focused on optimizing tissue acquisition strategies. In a systematic review and meta-analysis including 19 randomized controlled trials involving 3552 patients, Chalhoub et al. (2024) demonstrated that three EUS-FNB passes, compared with two, improved diagnostic accuracy (OR = 1.58; 95% CI: 1.20-2.09), specimen adequacy (OR = 1.97; 95% CI: 1.30-2.83), and diagnostic yield (OR = 2.12; 95% CI: 1.37-3.27). Additional fourth or fifth passes did not significantly improve diagnostic outcomes. In subgroup analyses involving contemporary Franseen and fork-tip needles, two passes provided better diagnostic performance than a single pass. These findings support an individualized sampling strategy according to needle design and specimen adequacy, rather than a universally fixed number of passes [29].

Furthermore, Termsinsuk et al. (2025) reported comparable pooled diagnostic sensitivities for histological analysis alone and combined cytohistological assessment of EUS-FNB specimens (90.5%, 95% CI: 85.8-94.2% versus 91.4%, 95% CI: 87.7-94.5%, respectively), based on 29 studies involving 1657 patients assessed by histological analysis and 1271 patients assessed by combined analysis. These findings suggest that high-quality core tissue may reduce the need for additional cytological processing in selected settings [30]. However, these pooled estimates should not be interpreted as demonstrating statistical equivalence between the two approaches. Further prospective studies are needed to confirm these findings [30].

Adequate tissue acquisition is especially relevant for non-ductal pancreatic neoplasms, which often require immunohistochemical confirmation of their cellular differentiation [1]. In PanNENs, EUS-guided sampling contributes to Ki-67 assessment and tumor grading, although intratumoral heterogeneity may result in discrepancies between biopsy and surgical specimens [31]. In PACC and SPN, tissue-based immunohistochemical analysis is particularly valuable when imaging and cytological findings overlap with those of other pancreatic neoplasms [11,25]. Overall, conventional and advanced EUS techniques improve lesion characterization, whereas EUS-guided tissue acquisition provides the diagnostic material required for accurate histological classification when imaging findings remain indeterminate [5].

Differential diagnosis of solid pancreatic neoplasms

The differential diagnosis of solid pancreatic neoplasms remains challenging because tumors arising from distinct histological lineages may exhibit overlapping morphological and enhancement characteristics [1,16]. An integrated diagnostic approach should therefore consider tumor growth pattern, vascularity, pancreatic duct involvement, and internal architecture, together with histopathological and immunohistochemical findings when imaging remains inconclusive [1].

Distinguishing Ductal From Non-ductal Pancreatic Neoplasms

PDAC typically demonstrates an infiltrative growth pattern, hypoenhancement, and upstream pancreatic duct dilatation, whereas non-ductal pancreatic neoplasms more frequently exhibit expansile growth and relatively well-defined margins [16,19]. Nevertheless, these morphological characteristics are not sufficiently specific to distinguish individual tumor subtypes, particularly when lesions demonstrate atypical enhancement or advanced local extension [18].

PACC represents an important diagnostic consideration because it may resemble PDAC when presenting as an ill-defined, hypovascular mass, or PanNETs when relatively circumscribed and showing increased vascularity [17,20]. Compared with PDAC, PACC more frequently exhibits well-defined margins, an exophytic growth pattern, and relatively limited pancreatic duct dilatation [32]. The presence of necrosis, heterogeneous enhancement, or limited ductal obstruction may suggest PACC, although none of these findings is pathognomonic [18,19].

Differentiating Acinar, Neuroendocrine, and Solid Pseudopapillary Neoplasms

PACC and well-differentiated PanNETs may share circumscribed margins and limited pancreatic duct involvement, whereas arterial hyperenhancement more commonly favors neuroendocrine differentiation [2,17]. However, higher-grade PanNETs and poorly differentiated pancreatic neuroendocrine carcinomas may exhibit heterogeneous or reduced enhancement, increasing their resemblance to other pancreatic malignancies [2].

SPNs may also resemble PanNETs, particularly when predominantly solid and lacking characteristic hemorrhagic or cystic degeneration [25]. Although a capsule, intratumoral hemorrhage, and heterogeneous or progressive enhancement may favor SPN, comparative imaging studies demonstrate considerable overlap between these entities [3]. The age and sex of the patient may provide additional diagnostic clues, as SPNs predominantly occur in young women, although they may also be encountered in other patient populations [13].

When imaging findings remain indeterminate, immunohistochemical analysis provides essential evidence of tumor differentiation [1]. Acinar differentiation is supported by pancreatic enzyme markers, particularly trypsin, chymotrypsin, and BCL10, whereas neuroendocrine differentiation is supported by synaptophysin, chromogranin A, and INSM1 [1,11]. Nuclear β-catenin expression, in conjunction with characteristic morphology, strongly supports SPN, although its interpretation requires consideration of potential immunophenotypic overlap with other pancreatic tumors [1,15].

An additional diagnostic challenge is the distinction between well-differentiated grade 3 PanNETs and poorly differentiated PanNECs, which relies primarily on morphology and may be supported by proliferative activity and alterations in p53 and Rb expression [1]. This distinction is clinically relevant because these tumors differ in their biological behavior, prognosis, and therapeutic management.

Rare Pancreatic Neoplasms and Secondary Pancreatic Tumors

Pancreatoblastoma is a rare pancreatic malignancy that may mimic PACC, particularly when presenting as a large, heterogeneous mass with necrotic or cystic components [1,6]. Although both neoplasms may demonstrate acinar differentiation, the identification of characteristic squamoid nests, sometimes associated with nuclear β-catenin expression, represents a key histopathological feature favoring pancreatoblastoma [6]. The younger age at presentation, particularly in children, may also contribute to the differential diagnosis [13].

Secondary pancreatic tumors constitute an important differential diagnosis, particularly in patients with a history of extrapancreatic malignancy [33]. Renal cell carcinoma is a prominent source of clinically recognized pancreatic metastases, while lung, colorectal, and breast carcinomas, as well as melanoma, may also involve the pancreas [33]. Pancreatic metastases from renal cell carcinoma typically demonstrate marked arterial hyperenhancement and may mimic well-differentiated pancreatic neuroendocrine tumors [34]. Their occurrence several years after nephrectomy highlights the importance of reviewing the patient's oncological history and obtaining histopathological confirmation when imaging findings are inconclusive [35].

Primary pancreatic lymphoma represents another uncommon diagnostic consideration and may present as a bulky, relatively homogeneous, hypovascular mass with limited pancreatic duct obstruction despite its size. Associated lymphadenopathy and vascular encasement without significant vascular obstruction may provide additional diagnostic clues [36]. Definitive diagnosis requires demonstration of lymphoid differentiation through histopathological and immunohistochemical evaluation, potentially supplemented by flow cytometry for accurate lymphoma subclassification [1]. The principal imaging characteristics, immunohistochemical profiles, and key diagnostic discriminators of these primary and secondary pancreatic neoplasms are summarized in Table 1 [1].

**Table 1:** Comparative imaging, immunohistochemical, and key diagnostic features of solid pancreatic neoplasms Immunohistochemical markers should be interpreted in conjunction with tumor morphology and clinical context. The listed imaging and immunohistochemical features represent characteristic patterns rather than absolute diagnostic criteria, and overlapping findings may occur among different tumor subtypes PDAC: pancreatic ductal adenocarcinoma; PACC: pancreatic acinar cell carcinoma; PanNET: pancreatic neuroendocrine tumor; PanNEC: pancreatic neuroendocrine carcinoma; SPN: solid pseudopapillary neoplasm; CT: computed tomography; MRI: magnetic resonance imaging; EUS: endoscopic ultrasound

Tumor type	CT/MRI and EUS findings	Typical immunohistochemical profile	Key diagnostic discriminator
PDAC	Ill-defined, infiltrative, hypovascular mass, upstream pancreatic duct dilatation, parenchymal atrophy, and possible vascular encasement [16]	CK7+, CK19+, MUC1+; aberrant p53 expression or SMAD4 loss in a subset [1]	Infiltrating malignant ductal glands within desmoplastic stroma; frequent ductal obstruction [1]
PACC	Often relatively well-circumscribed, expansile or exophytic mass; variable enhancement, heterogeneous areas of necrosis, occasional calcifications, and relatively infrequent ductal dilatation [17]	Trypsin+, chymotrypsin+, BCL10+; variable lipase expression; focal neuroendocrine marker expression may occur [11]	Acinar differentiation confirmed by pancreatic enzyme markers, particularly trypsin and BCL10 [1,11]
Well-differentiated PanNET	Usually well-defined and hypervascular; larger or higher-grade tumors may demonstrate heterogeneous or reduced enhancement [2]	Synaptophysin+, chromogranin A+, INSM1+; Ki-67 for grading [1]	Well-differentiated neuroendocrine morphology and Ki-67-based grading [31]
Poorly differentiated PanNEC	Often infiltrative and heterogeneous, with necrosis and variable enhancement [2]	Synaptophysin+, INSM1+; variable chromogranin A expression; aberrant p53 expression and/or Rb loss [1]	Small-cell or large-cell neuroendocrine carcinoma morphology, supported by immunohistochemical findings when distinguishing PanNEC from well-differentiated PanNET G3 [1]
SPN	Well-circumscribed, frequently encapsulated mass; variable solid-cystic composition, intratumoral hemorrhage, and heterogeneous enhancement [3]	Nuclear β-catenin+, CD10+, LEF1+; characteristic paranuclear dot-like CD99 staining; chromogranin A usually negative [15]	Nuclear β-catenin expression combined with characteristic pseudopapillary morphology supported by LEF1 expression and the distinctive CD99 staining pattern [1,15]
Pancreatoblastoma	Large, heterogeneous, expansile mass with possible necrotic or cystic components [6]	Trypsin+, BCL10+ in acinar areas; nuclear β-catenin may be present in squamoid nests [6]	Characteristic squamoid nests associated with acinar differentiation, often in pediatric patients [6]
Primary pancreatic lymphoma	Bulky, relatively homogeneous, hypovascular mass; limited ductal obstruction relative to tumor size; possible lymphadenopathy and vascular encasement without significant stenosis or occlusion [16]	CD45+; CD20+ in B-cell lymphomas; additional markers depend on lymphoma subtype [1]	Lymphoid morphology and immunophenotype; flow cytometry may assist subclassification [1]
Pancreatic metastases	Solitary or multiple lesions; enhancement varies according to the primary malignancy. Renal cell carcinoma metastases frequently demonstrate marked arterial hyperenhancement [34]	Depending on the primary tumor: PAX8+, CAIX+ (renal cell carcinoma); TTF-1+, Napsin A+ (pulmonary adenocarcinoma); CDX2+, SATB2+ (colorectal carcinoma); GATA3+, ER+ (breast carcinoma); SOX10+, Melan-A+ (melanoma). These markers should be interpreted as part of an appropriate immunohistochemical panel rather than individually [1]	Morphological and immunohistochemical concordance with an extrapancreatic primary tumor, correlation with oncological history [33,35]

Diagnostic Algorithm for Solid Pancreatic Neoplasms

To facilitate the clinical application of the findings summarized in this review, a practical diagnostic algorithm is proposed (Figure 1). This approach integrates clinical assessment, cross-sectional imaging, EUS, and histopathological evaluation when indicated. The predominant imaging phenotype provides an initial diagnostic orientation, while EUS and complementary techniques may further characterize indeterminate lesions. When tissue confirmation is required, EUS-FNB enables histological and immunohistochemical assessment. The proposed algorithm represents a synthesis of the available literature rather than a prospectively validated diagnostic pathway [1,4,5].

**Figure 1:**
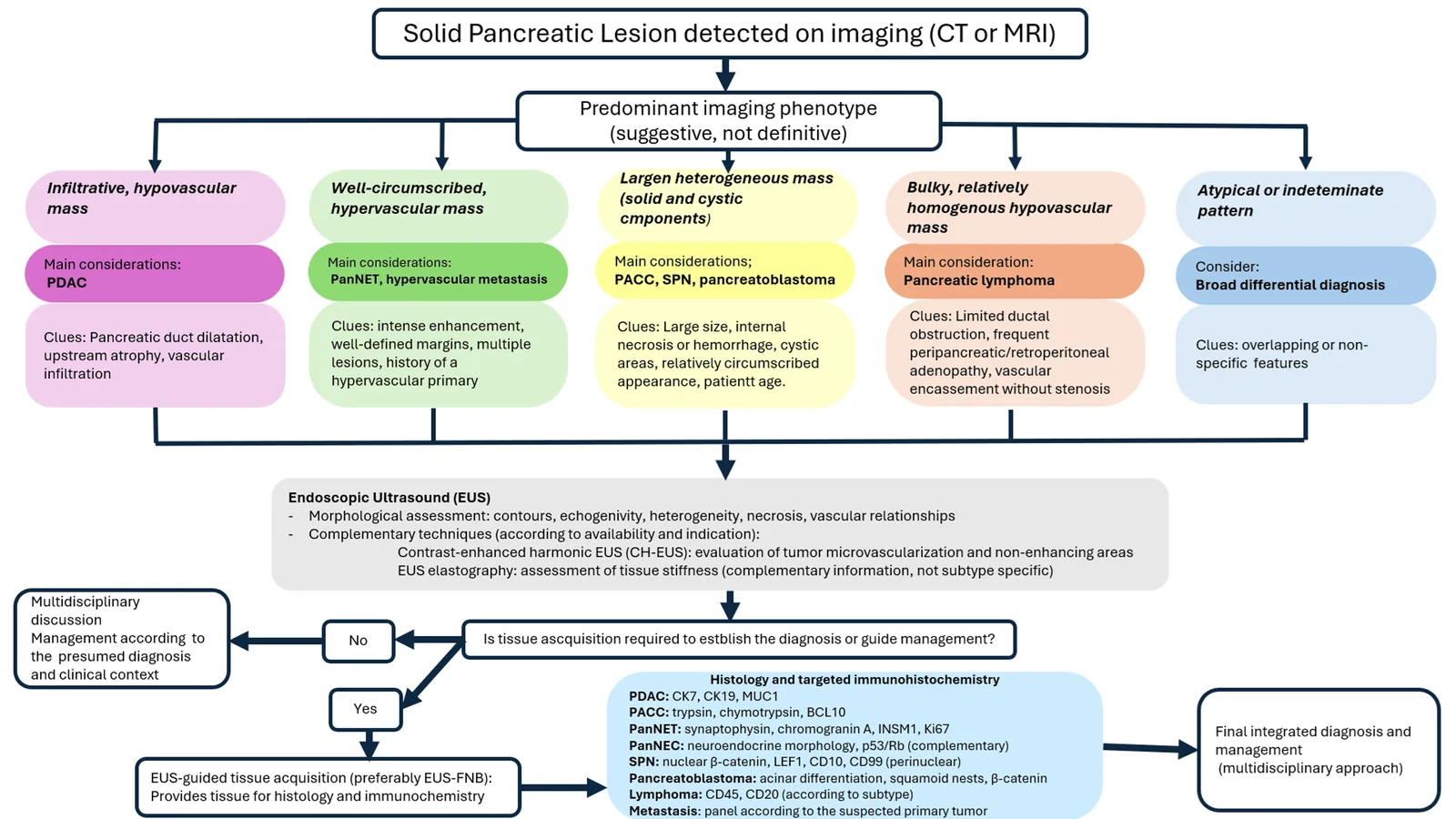
Proposed diagnostic algorithm for the differential diagnosis of solid pancreatic neoplasms CT: computed tomography; MRI: magnetic resonance imaging; EUS: endoscopic ultrasound; CH-EUS: contrast-enhanced harmonic endoscopic ultrasound; EUS-FNB: endoscopic ultrasound-guided fine needle biopsy; PDAC: pancreatic ductal adenocarcinoma; PACC: pancreatic acinar cell carcinoma; PanNET: pancreatic neuroendocrine tumor; SPN: solid pseudopapillary neoplasm; IHC: immunohistochemistry Image created by the authors

Discussion and future directions

The differential diagnosis of solid pancreatic neoplasms remains challenging because ductal and non-ductal tumors frequently share overlapping radiological and endosonographic features [2]. Although certain imaging patterns may suggest a specific histological subtype, accurate diagnosis requires a combined assessment of cross-sectional imaging, EUS, and histopathological findings when tissue confirmation is indicated [1]. EUS-FNB has improved the diagnostic evaluation of solid pancreatic lesions by providing adequate tissue samples for histological classification, immunohistochemistry, and selected molecular analyses. This is particularly relevant for uncommon neoplasms, including pancreatic acinar cell carcinoma, pancreatic neuroendocrine neoplasms, and SPNs, whose management and prognosis differ substantially from those of pancreatic ductal adenocarcinoma [1,11].

Emerging technologies, particularly radiomics and artificial intelligence (AI), may improve the characterization of pancreatic lesions by identifying quantitative imaging features beyond conventional visual assessment [37]. Recent studies investigating AI-assisted EUS and multimodal imaging approaches have reported promising results in pancreatic lesion characterization. However, their clinical application remains limited by the need for external validation, standardized imaging protocols, and prospective evaluation [38]. Nevertheless, evidence concerning rare pancreatic neoplasms remains limited by small cohorts, retrospective study designs, and heterogeneous imaging protocols [2,18]. These limitations may reduce the generalizability of reported imaging characteristics and diagnostic performance. Future multicenter studies integrating standardized imaging, advanced EUS techniques, and molecular pathology are needed to improve diagnostic accuracy and support individualized therapeutic strategies [11,37].

## Conclusions

Solid pancreatic neoplasms encompass a heterogeneous group of tumors with overlapping radiological and endosonographic features, which makes accurate preoperative differentiation challenging. Although CT, MRI, and EUS provide valuable diagnostic information, imaging findings alone are often insufficient to establish a definitive histological diagnosis. Recognizing characteristic imaging patterns and their limitations may help narrow the differential diagnosis between ductal and non-ductal pancreatic neoplasms. An integrated approach that combines cross-sectional imaging, EUS-guided tissue acquisition when indicated, and immunohistochemical analysis is particularly important when imaging findings remain inconclusive, especially for uncommon non-ductal pancreatic neoplasms. The comparative diagnostic framework and proposed algorithm presented in this review aim to support a structured, multidisciplinary approach to diagnostic decision-making. Advances in molecular pathology and emerging AI-based imaging techniques may further improve diagnostic precision and support more individualized patient management.

## References

[1] Wang Q, Reid MD (2022). Cytopathology of solid pancreatic neoplasms: an algorithmic approach to diagnosis. Cancer Cytopathol.

[2] Möller K, Löwe A, Jenssen C (2024). Comments and illustrations of the European Federation of Societies for Ultrasound in Medicine contrast-enhanced ultrasound guidelines. Rare pancreatic tumors, imaging features on transabdominal ultrasound and EUS with contrast enhancement: Rare epithelial pancreatic tumors: solid pseudopapillary neoplasm, acinar cell carcinoma, mixed neuroendocrine-non-neuroendocrine neoplasms, some rare subtypes of pancreatic adenocarcinoma and pancreatoblastoma. Endosc Ultrasound.

[3] Khristenko E, Gaida MM, Tjaden C (2024). Imaging differentiation of solid pseudopapillary neoplasms and neuroendocrine neoplasms of the pancreas. Eur J Radiol Open.

[4] Machicado JD, Sheth SG, Chalhoub JM (2024). American Society for Gastrointestinal Endoscopy guideline on the role of endoscopy in the diagnosis and management of solid pancreatic masses: summary and recommendations. Gastrointest Endosc.

[5] Facciorusso A, Arvanitakis M, Crinò SF (2025). Endoscopic ultrasound-guided tissue sampling: European Society of Gastrointestinal Endoscopy (ESGE) Technical and Technology Review. Endoscopy.

[6] Klimstra DS (2007). Nonductal neoplasms of the pancreas. Mod Pathol.

[7] Zheng C, Wang J, Wang J, Zhang Q, Liang T (2024). Cell of origin of pancreatic cancer: novel findings and current understanding. Pancreas.

[8] Luchini C (2026). Pancreatic ductal adenocarcinoma and its subtypes: clinical relevance of histopathology and molecular characterization, integrating the key updates of the 2026 WHO classification. Virchows Arch.

[9] La Rosa S, Maragliano R, Marchiori D (2025). Roadmap to challenges in limited cellularity specimens from pancreatic neuroendocrine neoplasms: diagnostic tools for the most appropriate management of limited material. Endocr Pathol.

[10] La Rosa S, Sessa F, Capella C (2015). Acinar cell carcinoma of the pancreas: overview of clinicopathologic features and insights into the molecular pathology. Front Med (Lausanne).

[11] Ikezawa K, Urabe M, Kai Y, Takada R, Akita H, Nagata S, Ohkawa K (2024). Comprehensive review of pancreatic acinar cell carcinoma: epidemiology, diagnosis, molecular features and treatment. Jpn J Clin Oncol.

[12] Kim JY, Brosnan-Cashman JA, Kim J (2020). Pancreatic acinar cell carcinomas and mixed acinar-neuroendocrine carcinomas are more clinically aggressive than grade 1 pancreatic neuroendocrine tumours. Pathology.

[13] Omiyale AO (2021). Solid pseudopapillary neoplasm of the pancreas. World J Hepatol.

[14] Zalatnai A, Kis-Orha V (2020). Solid-pseudopapillary neoplasms of the pancreas is still an enigma: a clinicopathological review. Pathol Oncol Res.

[15] Liu Y, Peng J, Zhao Y, Wang W (2025). Emerging pathological diagnostic strategies for solid pseudopapillary neoplasm of the pancreas: insights from omics and innovative techniques. J Pathol Clin Res.

[16] Shah S, Mortele KJ (2007). Uncommon solid pancreatic neoplasms: ultrasound, computed tomography, and magnetic resonance imaging features. Semin Ultrasound CT MR.

[17] Qu Q, Xin Y, Xu Y, Yuan Y, Deng K (2022). Imaging and clinicopathological features of acinar cell carcinoma. Front Oncol.

[18] Calimano-Ramirez LF, Daoud T, Gopireddy DR (2022). Pancreatic acinar cell carcinoma: a comprehensive review. World J Gastroenterol.

[19] Tatli S, Mortele KJ, Levy AD, Glickman JN, Ros PR, Banks PA, Silverman SG (2005). CT and MRI features of pure acinar cell carcinoma of the pancreas in adults. AJR Am J Roentgenol.

[20] Jornet D, Soyer P, Terris B (2019). MR imaging features of pancreatic acinar cell carcinoma. Diagn Interv Imaging.

[21] Zignani N, Balzarini M, Dabizzi E (2025). Endoscopic ultrasound features of pancreatic solid lesions: descriptive and predictive analysis on a multicenter sample. World J Gastrointest Endosc.

[22] Yamashita Y, Shimokawa T, Ashida R (2022). Comparison of endoscopic ultrasonography with and without contrast enhancement for characterization of pancreatic tumors: a meta-analysis. Endosc Int Open.

[23] Yao DW, Qin MZ, Jiang HX, Qin SY (2024). Comparison of EUS-FNA and EUS-FNB for diagnosis of solid pancreatic mass lesions: a meta-analysis of prospective studies. Scand J Gastroenterol.

[24] Chantarojanasiri T, Hirooka Y, Kawashima H (2016). Endoscopic ultrasound in the diagnosis of acinar cell carcinoma of the pancreas: contrast-enhanced endoscopic ultrasound, endoscopic ultrasound elastography, and pathological correlation. Endosc Int Open.

[25] Pawlak KM, Tehami N, Maher B (2023). Role of endoscopic ultrasound in the characterization of solid pseudopapillary neoplasm of the pancreas. World J Gastrointest Endosc.

[26] Yamashita Y, Kitano M (2024). Role of contrast-enhanced harmonic endoscopic ultrasonography (EUS) and EUS elastography in pancreatic lesions. Clin Endosc.

[27] Kataoka K, Ishikawa T, Ohno E (2022). Differentiation between solid pseudopapillary neoplasm of the pancreas and nonfunctional pancreatic neuroendocrine neoplasm using endoscopic ultrasound. Pancreas.

[28] Wang F, Ma J, Liu H (2025). Endoscopic ultrasound elastography in solid pancreatic lesions: individual and combined performance of strain ratio, fat-lesion strain ratio, and mean strain histogram for diagnosing pancreatic ductal adenocarcinoma. Clin Exp Med.

[29] Chalhoub JM, Hawa F, Grantham T (2024). Effect of the number of passes on diagnostic performance of EUS fine-needle biopsy of solid pancreatic masses: a systematic review and meta-analysis. Gastrointest Endosc.

[30] Termsinsuk P, Chuncharunee A, Charatcharoenwitthaya P, Pausawasdi N (2025). Diagnostic performance of endoscopic ultrasound-guided fine needle biopsy with histological analysis versus combined cytohistological analysis in solid pancreatic lesions: a systematic review and meta-analysis. Dig Dis Sci.

[31] Pyo JS, Kim NY, Min KW, Oh IH, Lim DH, Son BK (2023). Diagnostic accuracy of ki-67 labeling index in endoscopic ultrasonography-fine-needle aspiration cytology and biopsy of pancreatic neuroendocrine neoplasms. Diagnostics (Basel).

[32] Barat M, Dohan A, Gaujoux S (2020). Computed tomography features of acinar cell carcinoma of the pancreas. Diagn Interv Imaging.

[33] Adsay NV, Andea A, Basturk O, Kilinc N, Nassar H, Cheng JD (2004). Secondary tumors of the pancreas: an analysis of a surgical and autopsy database and review of the literature. Virchows Arch.

[34] Vincenzi M, Pasquotti G, Polverosi R, Pasquali C, Pomerri F (2014). Imaging of pancreatic metastases from renal cell carcinoma. Cancer Imaging.

[35] Balaban DV, Coman L, Marin FS (2023). Metastatic renal cell carcinoma to pancreas: case series and review of the literature. Diagnostics (Basel).

[36] Segaran N, Sandrasegaran K, Devine C, Wang MX, Shah C, Ganeshan D (2021). Features of primary pancreatic lymphoma: a bi-institutional review with an emphasis on typical and atypical imaging features. World J Clin Oncol.

[37] Podină N, Gheorghe EC, Constantin A (2025). Artificial intelligence in pancreatic imaging: a systematic review. United European Gastroenterol J.

[38] Zhang C, Tao X, Zhang J (2025). A multimodal artificial intelligence system for the detection and diagnosis of solid pancreatic lesions under EUS. Endosc Ultrasound.

